# Correlates of Objectively Measured Physical Activity Among People With Multiple Sclerosis: A Cross-Sectional Study

**DOI:** 10.3389/fresc.2021.726436

**Published:** 2021-12-10

**Authors:** Jennifer Fortune, Meriel Norris, Andrea Stennett, Cherry Kilbride, Grace Lavelle, Wendy Hendrie, Lorraine DeSouza, Christina Victor, Jennifer Mary Ryan

**Affiliations:** ^1^Ageing Studies Theme, Institute of Environment, Health and Societies, Brunel University London, London, United Kingdom; ^2^Multiple Sclerosis (MS) Therapy Centre, Norwich, United Kingdom

**Keywords:** multiple sclerosis, light physical activity (LPA), moderate to vigorous physical activity (MVPA), physical activity, MS

## Abstract

**Background:** Identifying correlates of physical activity (PA) for people with multiple sclerosis (MS) is essential to design effective PA interventions.

**Methods:** Participants completed a battery of questionnaires and wore an ActiGraph accelerometer. Light physical activity (LPA) and moderate-to-vigorous physical activity (MVPA) (min/day) were calculated. Associations were examined using multiple linear regression adjusted for demographic and clinical confounders.

**Results:** Fifty-eight adults with MS participated (mean ± SD age: 56.8 ± 9.2 yr; 67% women). MS type was associated with time in LPA. Participants with secondary progressive MS (*B* = −54.0, 95% CI −84.7 to −23.3) and primary progressive MS (*B* = −42.9, 95% CI −77.5 to −8.3) spent less time in LPA than those with relapsing remitting MS. Walking capacity, assessed using the 12-item MS walking scale (MSWS-12), was associated with time in MVPA (*B* = −0.36, 95% CI −0.72 to −0.01).

**Conclusion:** This work identifies walking capacity and type of MS as correlates of PA, which may indicate development of interventions to promote PA.

## Introduction

Increasing physical activity (PA) represents a safe (1) and cost-effective (2) approach for managing the sequelae of multiple sclerosis (MS) (3). PA is positively associated with walking mobility (4), quality of life (5), depression and fatigue (6), and cardiovascular health (7). Despite these benefits, physical inactivity is common. People with MS have lower step counts (8) and engage in significantly less moderate-to-vigorous physical activity (MVPA) than the general population (8, 9). Between 60% (10) and 80% of people with MS do not meet the minimum recommended PA volumes (9).

Identifying modifiable variables that are associated with PA may establish targets to support changes in PA and indicate the development of programmes to promote PA among people with MS (11). Further, establishing non-modifiable demographic and clinical correlates of PA may inform decisions on which subgroups of people with MS may particularly benefit from PA intervention. A systematic review by Streber et al. (12) identified that employment status, education level, disability level, walking limitations, and self-efficacy are consistently correlated with PA in people with MS. Age, BMI, falls history, type of MS, fatigue, and depression were inconsistently associated with PA (12). Inconsistent findings between studies may in part be attributable to limited consideration of possible confounding factors such as disability status. Further, the review identified that findings were limited by the use of self-report measures of PA (12). Studies published since the review continue to apply self-report PA measures (13–17). As self-report measures of PA demonstrate poor agreement with objective measures of PA such as accelerometers (18), employing objective measures is important to ensure accurate quantification of PA while examining its correlates.

This work, therefore aimed to address limitations of previous research by examining modifiable and non-modifiable correlates of objectively measured light PA (LPA) and MVPA in ambulatory adults with MS. A second aim was to examine if associations were modified by disability status, as measured by the expanded disability status scale (EDSS).

## Methods

This cross-sectional study presents an analysis of baseline data from the iStep-MS trial. The iStep-MS trial was a feasibility randomized controlled trial of a behavior change intervention, which aimed to increase PA and reduce sedentary behavior in people with MS (19).

### Participants

Sixty people with MS were recruited from an MS Therapy Center in England and the MS Society UK website. Inclusion criteria were: a self-reported diagnosis of MS, self-reported relapse-free for the past 3 months, free of unstable medical conditions such as unstable angina that would make it unsafe to participate in PA, and ability to independently walk within the home with or without a walking aid. Exclusion criteria were pregnancy and ongoing participation in other trials. Written informed consent was obtained from all participants. The College of Health and Life Sciences Research Ethics Committee in Brunel University London (6181-NHS-Apr/2017-7016-2) approved this work.

### Independent Variables

We included demographic and clinical characteristics as independent variables in this analysis. Participants completed a questionnaire at baseline that provided information on their age, sex, ethnicity, living arrangement (i.e., living alone or living with family/partner), employment status, marital status, type of MS, and duration of MS. Participants could request support from the researcher to complete the questionnaire if required. Participants were categorized as EDSS (20) levels 1.0–4.0 or 4.5–6.5. A researcher measured participants' weight using a Seca 875 Flat Scale, height using a Seca 213 portable stadiometer, and then calculated body mass index (BMI). Waist circumference was measured using a tape (Seca) on bare skin, to the nearest 0.1 cm midway between the lower rib margin and the iliac crest at the end of gentle expiration. The mean of two measurements was used for waist circumference.

Fatigue was assessed using the modified fatigue impact scale (MFIS). Self-efficacy was assessed using the multiple sclerosis self-efficacy scale (MSSE) function and control subscales. Walking capability was assessed using the 12-item MS walking scale (MSWS-12). The physical and psychological impact of MS was assessed using the multiple sclerosis impact scale (MSIS-29). Health-related quality of life (HRQOL) was assessed using EuroQol-5D-5L (21). The United Kingdom value set was used to calculate a utility score (22). Participation over four domains (autonomy indoors, family role, autonomy outdoors, and social life and relationships) was assessed using the impact on participation and autonomy questionnaire (IPA). The median score was obtained for each participant for each subscale. A full description of the measurement of these variables measured is provided elsewhere (23). Variable scoring is outlined in Supplementary Table 1.

### Dependent Variables

Time in light PA and MVPA were included as dependent variables in this analysis. Participants were asked to wear an ActiGraph wGT3X-BT accelerometer (ActiGraph, Pensacola, Florida, USA) for 7 days. The ActiGraph was worn on an elastic belt at the mid-axillary line at the hip during waking hours only and removed for any water-based activities. Non-wear-time was defined as ≥90 consecutive minutes of 0 counts (24) and was validated against wear-time diaries kept by participants. Days with ≥10 h of wear data were considered valid and participants with at least 3 valid days were included in analysis (25). LPA was determined using a threshold of ≥100 counts per minute and <1,745 counts per minute. MVPA was classified as ≥1,745 counts per minute using established MS specific cut points (26).

### Data Analysis

Statistical analysis was performed using Stata, version 16.0 (StataCorp LP, College Station, Texas). The distribution of data was examined using histograms, Q–Q plots, and cross-tabulations. Data are summarized as mean, standard deviation, median, minimum, maximum, frequencies, and proportions as appropriate. Separate linear regression models were used to examine unadjusted associations between demographic and clinical characteristics (included as independent variables), and LPA and MVPA (included as dependent variables), respectively. All demographic and clinical characteristics that were associated with LPA at the level of *p* < 0.05 were included in a multiple linear regression model. Similarly, characteristics associated with MVPA at the level of *p* < 0.05 were included in a multiple linear regression model. Where we observed an association between a characteristic and LPA or MVPA, we included an interaction term between the characteristic and EDSS category to examine if the association was modified by disability status. Assumptions of linear regression were assessed by visually inspecting Q–Q plots of residuals and scatter plots of residuals against fitted values. There was no evidence of heteroscedasticity or non-normally distributed residuals.

## Results

Two participants did not return the accelerometer, resulting in 58 participants included in the analysis. Table 1 displays the demographic and clinical characteristics of included participants. Participants had a mean (standard deviation [SD]) age of 56.8 (9.2) years, and were predominantly women (67%) and white (88%). Most (86%) lived with a partner, spouse, or family member, and 14% lived alone. Seventy-eight per cent were married/partnered and 13% were not married/partnered. Sixty-seven per cent were not in paid employment, 33% were in paid employment. Most (74%) were in EDSS 4.5–6.5, and 26% were in EDSS 1.0–4.0. Approximately a third of participants had relapsing-remitting MS, 36% had secondary progressive MS, and 22% had primary progressive MS. Median duration since diagnosis of MS was 13 years (range 1–42 years).

**Table 1:** Participant characteristics.

	**n (%)**	**Mean (SD)**	**Median (range)**
Age, years	58	56.8 (9.2)	57 (37-74)
Female	39 (67)		
**Ethnicity**					
White	51 (88)		
Black	4 (7)		
Asian	3 (5)		
**Living arrangement**					
Lives alone	8 (14)		
Lives with partner/spouse/family	50 (86)		
**Employment status**					
In paid employment	19 (33)		
Not in paid employment	39 (67)		
**Marital status**					
Married/partnered	45 (78)		
Not married/partnered	13 (22)		
BMI, kg.m^2^	58	26.2 (5.5)	24.29 (16.77–47.99)
Waist circumference, cm	58	96.4 (15.2)	95.8 (70–154.2)
MS duration, years	57	15.4 (9.8)	13 (1–42)
**Type of MS**					
Relapsing-remitting	20 (35)		
Secondary progressive	21 (36)		
Primary progressive	13 (22)		
Unknown	4 (7)		
**EDSS**					
1.0–4.0	15 (26)		
4.5–6.5	43 (74)		
MFIS (0–84)	58	43.2 (18.4)	44 (1–81)
MMSE control (90–900)	58	573.3 (201.2)	570 (230–890)
MMSE function (90–900)	58	661.2 (197.6)	690 (180–900)
MSWS-12, %	58	74.6 (20.0)	79.2 (20.0–100.0)
MSIS-29 physical (0–100)	58	43.0 (21.3)	42.5 (3.8–86.3)
MSIS-29 psychological (0–100)	58	31.6 (20.1)	30.6 (0.0–86.1)
EQ-5D-5L utility	58	0.63 (0.19)	0.64 (-0.04 to 1.00)
IPA: autonomy indoors (0–4)	58	0.67 (0.87)	0 (0–3)
IPA: family role (0–4)	58	1.33 (0.94)	1 (0–3)
IPA: autonomy outdoors (0–4)	58	1.57 (1.11)	1 (0–4)
IPA: social life and relationships (0–4)	58	0.48 (0.60)	0 (0–2)

*BMI, body mass index; EDSS, Expanded Disability Status Scale; IPA, Impact on Participation and Autonomy Questionnaire; MFIS, Modified Fatigue Impact Scale; MS, multiple sclerosis; MSSE, Multiple Sclerosis Self-Efficacy Scale; MSWS-12, Twelve Item MS Walking Scale; SD, standard deviation. MS duration n = 1 missing*.

Participants wore the accelerometer for a mean (SD) 6.77 (0.83) days and 851.20 (84.61) min/day (range 658–1092.16 min/day). Time spent in LPA and MVPA is described in Table 2.

**Table 2:** Time spent in light and moderate-to-vigorous activity (*n* = 58).

	**Mean (SD)**	**Median (IQR)**
Light PA (min/day)	157.74 (52.64)	159.12 (125.96–192.32)
Moderate-to-vigorous PA (min/day)	17.93 (16.21)	11.68 (5.07–29.14)

*IQR, interquartile range; PA, physical activity; SD, standard deviation*.

Table 3 presents the unadjusted and adjusted associations between demographic and clinical characteristics, and time in LPA. In unadjusted analyses, women spent on average 34.6 min (95% CI 6.3–62.9 min) more than men in LPA per day. Asian participants spent on average 63.4 min (95% CI 2.4–124.3 min) less than white participants in LPA per day. People with secondary progressive and primary progressive MS spent less time in LPA than people with relapsing remitting MS (coeff. −56.6, 95% CI −85.9 to −27.4, and coeff. −53.5, 95% CI −86.8 to −20.1, respectively). The MMSE function subscale and EQ-5D-5L utility score were positively associated with time in LPA (coeff. 0.10, 95% CI 0.03–0.16, and coeff. 101.4, 95% CI 32.0–170.7, respectively). The MSIS-29 physical subscale was negatively associated with time in LPA (coeff. −0.68, 95% CI −1.32 to −0.05).

**Table 3:** Associations between demographic, clinical characteristics, and light physical activity.

	**LPA (min/day)**		**LPA (min/day)**	
	**Unadjusted Coeff. (95% CI)**	***p***	**Adjusted Coeff. (95% CI)**	***p***
Age, years	−1.32 (−2.81 to 0.18)	0.084	-	
Female (ref: male)	34.6 (6.3 to 62.9)	0.017	20.27 (−6.84 to 47.38)	0.139
**Ethnicity (ref: White)**				
Black	−29.6 (−82.9 to 23.6)	0.270	−27.2 (−76.4 to 21.9)	0.271
Asian	−63.4 (−124.3 to −2.4)	0.042	−62.0 (−127.0 to 2.9)	0.061
Living alone (ref: living with partner/spouse/family)	−16.9 (−57.1 to 23.4)	0.405	-	
Not in paid employment (ref: in paid employment)	−20.0 (−49.3 to 9.3)	0.177	-	
Not married/partnered (ref: married/partnered)	−0.67 (−34.2 to 32.8)	0.968	-	
BMI, kg.m^2^	−1.75 (−4.26 to 0.76)	0.168	-	
Waist circumference, cm	−0.62 (−1.53 to 0.29)	0.179	-	
MS duration, years	−1.22 (−2.64 to 0.20)	0.092	-	
**Type of MS (ref: Relapsing-remitting)**				
Secondary progressive	−56.6 (−85.9 to −27.4)	<0.000	−54.0 (−84.7 to −23.3)	0.001
Primary progressive	−53.5 (−86.8 to −20.1)	0.002	−42.9 (−77.5 to −8.3)	0.016
Unknown	−14.4 (−65.7 to 36.8)	0.575	−21.7 (−73.1 to 29.7)	0.400
EDSS 4.5-6.5 (ref: EDSS 1.0-4.0)	−25.1 (−56.3 to 6.1)	0.112	–	
MFIS (0–84)	−0.26 (−1.02 to 0.51)	0.506	-	
MMSE function (90–900)	0.10 (0.03 to 0.16)	0.005	−0.01 (−0.12 to 0.10)	0.849
MMSE control (90–900)	0.05 (−0.02 to 0.12)	0.125	-	
MSWS-12, %	−0.66 (−1.34 to 0.02)	0.057	-	
MSIS-29 psychological (0–100)	−0.32 (−1.02 to 0.37)	0.355	-	
MSIS-29 physical (0–100)	−0.68 (−1.32 to −0.05)	0.036	0.26 (−0.69 to 1.22)	0.583
EQ-5D-5L utility	101.4 (32.0 to 170.7)	0.005	76.8 (−17.2 to 170.7)	0.107
IPA: autonomy indoors (0–4)	−19.1 (−34.5 to −3.6)	0.017	6.88 (−14.2 to 27.9)	0.514
IPA: family role (0–4)	−12.4 (−26.9 to 2.2)	0.094	-	
IPA: autonomy outdoors (0–4)	−10.3 (−22.7 to 2.1)	0.103	-	
IPA: social life and relationships (0–4)	−29.9 (−52.0 to −7.8)	0.009	−16.6 (−42.5 to 9.3)	0.204

*BMI, body mass index; EDSS, Expanded Disability Status Scale; IPA, Impact on Participation and Autonomy Questionnaire; MFIS, Modified Fatigue Impact Scale; MS, multiple sclerosis; MSSE, Multiple Sclerosis Self-Efficacy Scale; MSWS-12, Twelve Item MS Walking Scale; SD, standard deviation*.

In the multiple linear regression model, only type of MS was associated with time in LPA. Specifically, people with secondary progressive MS spent on average 54 min (95% CI −84.7 to 23.3 min) less in LPA per day than those with relapsing remitting MS. People with primary progressive MS also spent on average 42.9 min (95% CI −77.5 to −8.3 min) less in LPA per day than people with relapsing remitting MS. There was no evidence that the association between type of MS and LPA differed in those with EDSS score 1.0–4.0 compared with those with EDSS score 4.5–6.5. When an MS type-by-EDSS interaction term was included in the multiple linear regression, there was no evidence that the association between type of MS and LPA was different between people with EDSS levels 1.0–4.0 and those in levels 4.5–6.5 (*p* = 0.565).

Table 4 presents the associations between demographic and clinical characteristics and time in MVPA. In unadjusted analyses, women spent more time in MVPA than men (coeff. 8.79, 95% CI −0.07 to 17.65). People who were not in paid employment spent less time in MVPA than those in paid employment (coeff. −16.9, 95% CI −24.8 to −8.9). People with secondary progressive MS and primary progressive MS spent less time in MVPA than those with relapsing remitting MS (coeff. −17.3, 95% CI −26.2 to −8.3, and coeff. −18.3, 95% CI −28.5 to −8.10, respectively). People with EDSS 4.5–6.5 spent less time in MVPA than those with 1.0–4.0 (coeff. −18.9, 95% CI −27.3 to −10.5). Age (coeff. −0.59, 95% CI −1.04 to −0.14), MS duration (coeff. −0.60, 95% CI −1.02 to −0.18), MFIS (coeff. −0.49, 95% CI −0.67 to −0.32), MSWS-12 (coeff. −0.49, 95% CI −0.67 to −0.32), and MSIS-29 physical subscale (coeff. −0.33, 95% CI −0.51 to −0.15) were negatively associated with time in MVPA. MMSE function subscale (coeff. 0.03, 95% CI 0.01 to 0.05), MMSE control subscale (coeff. 0.02, 95% CI 0.00 to 0.05), and EQ-5D-5L utility score (coeff. 28.9, 95% CI 7.3 to 50.5) were positively associated with time in MVPA. The IPA family role subscale (coeff. −5.07, 95% CI −9.47 to −0.68), autonomy outdoors subscale (coeff. −4.94, 95% CI −8.62 to −1.26), and social life and relationship subscale (coeff. −7.65, 95% CI −14.59 to −0.70) were negatively associated with time in MVPA.

**Table 4:** Associations between demographic, clinical characteristics, and moderate-to-vigorous physical activity.

	**MVPA (min/day)**		**MVPA (min/day)**	
	**Unadjusted Coeff. (95% CI)**	***p***	**Adjusted Coeff. (95% CI)**	***p***
Age, years	−0.59 (−1.04 to −0.14)	0.011	0.04 (−0.52 to 0.60)	0.887
Female (ref: male)	8.79 (−0.07 to 17.65)	0.052	-	
**Ethnicity (ref: White)**				
Black	−8.34 (−25.27 to 8.58)	0.327	-	
Asian	−8.25 (−27.63 to 11.11)	0.397	-	
Living alone (ref: living with partner/spouse/family)	−6.16 (−18.53 to 6.20)	0.322	-	
Not in paid employment (ref: in paid employment)	−16.9 (−24.8 to −8.9)	<0.000	−2.59 (−14.34 to 9.17)	0.659
Not married/partnered (ref: married/partnered)	−5.05 (−15.28 to 5.18)	0.327	-	
BMI, kg.m^2^	0.09 (−0.70 to 0.87)	0.820	-	
Waist circumference, cm	−0.05 (−0.33 to 0.24)	0.731	-	
MS duration, years	−0.60 (−1.02 to −0.18)	0.006	−0.39 (−0.90 to 0.12)	0.126
**Type of MS (ref: relapsing-remitting)**				
Secondary progressive	−17.3 (−26.2 to −8.3)	<0.000	−6.63 (−16.95 to 3.69)	0.202
Primary progressive	−18.3 (−28.5 to −8.10)	0.001	−10.53 (−23.88 to 2.81)	0.118
Unknown	−8.0 (−23.6 to 7.7)	0.314	−6.56 (−24.94 to 11.83)	0.475
EDSS 4.5-6.5 (ref: EDSS 1.0-4.0)	−18.9 (−27.3 to −10.5)	<0.000	−5.23 (−16.82 to 6.36)	0.367
MFIS (0–84)	−0.27 (−0.50 to −0.05)	0.019	0.05 (−0.32 to 0.42)	0.778
MMSE function (90–900)	0.03 (0.01 to 0.05)	0.004	−0.01 (−0.05 to 0.02)	0.470
MMSE control (90–900)	0.02 (0.00 to 0.05)	0.020	0.00 (−0.04 to 0.04)	0.979
MSWS-12, %	−0.49 (−0.67 to −0.32)	<0.000	−0.36 (−0.72 to −0.01)	0.047
MSIS-29 psychological (0–100)	−0.18 (−0.39 to 0.03)	0.086	-	
MSIS-29 physical (0–100)	−0.33 (−0.51 to −0.15)	0.001	0.13 (−0.29 to 0.54)	0.549
EQ-5D-5L utility	28.9 (7.3 to 50.5)	0.010	20.1 (−11.9 to 52.0)	0.211
IPA: autonomy indoors (0–4)	−4.78 (−9.62 to 0.06)	0.053	-	
IPA: family role (0–4)	−5.07 (−9.47 to −0.68)	0.024	0.80 (−4.96 to 6.57)	0.780
IPA: autonomy outdoors (0–4)	−4.94 (−8.62 to −1.26)	0.009	−1.55 (−7.83 to 4.73)	0.621
IPA: social life and relationships (0–4)	−7.65 (−14.59 to −0.70)	0.031	−1.71 (−9.72 to 6.30)	0.668

*BMI, body mass index; EDSS, Expanded Disability Status Scale; IPA, Impact on Participation and Autonomy Questionnaire; MFIS, Modified Fatigue Impact Scale; MS, multiple sclerosis; MSSE, Multiple Sclerosis Self-Efficacy Scale; MSWS-12, Twelve Item MS Walking Scale; SD, standard deviation*.

In the adjusted model, only MSWS-12 was associated with time in MVPA. A 1% increase in MSWS-12 was associated with, on average, a decrease of 0.36 min of MVPA per day (95% CI −0.72 to −0.01 min/day). There was evidence that the association between MSWS-12 and MVPA differed depending on EDSS score, as indicated by the *p*-value for the EDSS-by-MSWS interaction term (*p* = 0.028). For those with an EDSS score of between 1.0 and 4.0, a 1% increase in MSWS-12 was associated with a decrease of 0.57 min of MVPA per day (95% CI −0.95 to −0.18; *p* = 0.005). However, there was no association between MSWS-12 and MVPA for those with an EDSS score of between 4.5 and 6.5 (coeff. −0.03, 95% CI −0.48 to 0.42, *p* = 0.878).

## Discussion

This study examined modifiable and non-modifiable correlates of accelerometer-determined LPA and MVPA in a sample of adults with MS. In the adjusted regression model, type of MS was associated with LPA, and walking capability as measured by the MSWS-12 was associated with MVPA.

In line with previous research, inverse relationships between MS duration (9), disability status (27–29), age (30), and MVPA were demonstrated in the unadjusted analyses. Being a woman and White were associated with higher levels of LPA. Recent research demonstrated that men with MS exhibit higher levels of LPA than women (31, 32). However, a review concluded that sex is inconsistently associated with PA (12). Disagreement may be explained by differences between studies in terms of the PA construct examined, disability level, or personal characteristics (e.g., self-efficacy) of the sample (12).

Two studies have examined the association between PA and ethnicity among people with MS. One found no difference in objectively measured MVPA between White people and people from other ethnic backgrounds (9), and the second found a difference in self-reported PA between Black and White participants (33). To our knowledge, this is the first study to examine the association between LPA and ethnicity. The high proportion of White participants limits this finding and exploration of PA participation, and influences of PA among individuals with MS from Black and Asian ethnic backgrounds is warranted. Further, the relatively low proportion of Black and Asian participants does reflect existing exercise (34) and PA (9) literature in people with MS, which is predominately composed of White participants, and highlights a need to identify how to engage and promote inclusion of people with MS from other ethnic backgrounds in similar studies.

No non-modifiable factor remained associated with MVPA in adjusted analyses. Only type of MS remained associated with LPA in adjusted analyses. People with secondary progressive and primary progressive MS spent on average 54 and 43 min per day less in LPA, respectively, than those with relapsing–remitting MS, even after controlling for sex, ethnicity, self-efficacy for function, physical impact of MS, quality of life, and participation and autonomy. Type of MS has been shown to be associated with objectively measured step count when controlling for age, cane use, number of years since MS diagnosis, employment status, and type of MS (30). Interventions for changing PA behavior in people with MS have predominantly included ambulatory participants with relapsing–remitting MS (35). The present results emphasize the need to provide interventions that promote PA to people with progressive disease courses.

In terms of potentially modifiable factors, employment status and fatigue were negatively associated with MVPA in the unadjusted analyses. Our findings align with previous research that demonstrates that unemployment (9) and fatigue (17) are negatively associated with PA.

In this work, both the EQ-5D-5L utility score and the MSIS-29 physical subscale correlated significantly with time in LPA and MVPA. These findings support cross-sectional research that demonstrated a positive association between quality of life and objective PA (36) and longitudinal studies that demonstrate alterations in PA yield favorable changes in physical and psychological disease impact (36). The IPA subscales were negatively associated with MVPA and LPA. This aligns with previous research in people with MS, which demonstrated poorer autonomy and participation in those with lower aerobic capacity (37). Experiences of participation and autonomy appear to be closely associated with perceived quality of life and disease impact (38). Focusing on strategies to enhance quality of life like social support (39) and assessment, and modification of environmental barriers which have a large and negative effect on participation (38, 40) in people with MS may influence these factors, and, in turn, positively influence PA.

Self-efficacy for function (i.e., confidence in performing behaviors associated with engaging in daily living activities) was positively associated with LPA and MVPA, and self-efficacy for control (i.e., confidence to manage disease symptoms, reactions, and impact on daily activities) was associated with MVPA. Self-efficacy is a consistent positive correlate of PA (12). Comparison with existing research is difficult due to varied PA data collection methods, examination of associations using univariable analyses (27, 41), or analyses that control for a wide range of confounding variables from environmental factors (16) to social cognitive theory constructs (42). In this work the relationship between self-efficacy and PA may have been confounded by the inclusion of MSWS-12 in the model which is negatively associated with both self-efficacy (43) and PA (44).

Walking capacity was the only independent predictor of MVPA in the adjusted analyses. No potentially modifiable factors remained associated with LPA. The negative association between MSWS-12 and MVPA in the adjusted analysis reflects previous research which demonstrated that more severe walking impairment is associated with reduced step count after controlling for disease duration and severity (45). Walking capacity fluctuates regularly across the disease course, even in those with relatively stable disease (46). Targeting interventions to improve walking capacity through for example core stability and balance (47) may represent a mechanism to help improve PA.

In this work, EDSS score significantly moderated the relationship between walking ability and MVPA. Walking capacity was associated with PA for participants with EDSS score 1.0–4.0. In participants with EDSS score > 4.5 no association between walking capacity and PA was demonstrated. It is possible that in participants with EDSS 1.0–4.0 there was sufficient variation in walking capacity and PA to show an association, whereas for people in EDSS 4.5–6.5 variation in walking capacity and PA was too limited to find an association. Strategies to improve walking capacity may be particularly beneficial for increasing PA in people with lower EDSS scores. However, interventions that focus on the types of activity other than walking, including resistance training and adapted exercise modalities such as electrical stimulation cycling (48), may promote more sustainable PA for individuals with higher levels of disability or mobility limitations.

### Strengths and Limitations

This study addressed limitations of previous research by using an objective PA measure and including a more diverse representation of people with both relapsing–remitting and progressive MS, rather than relapsing–remitting only. However, most participants were women and White, which limits the generalisability of results. Furthermore, as participants were recruited from an MS Center and the MS Society website they may be more engaged with PA than the general MS population, and therefore more motivated to take part in PA.The cross-sectional nature of this research precludes any inferences of causality. Finally the small sample size is a limitation.

### Implications

In summary, the findings of this study add to previous research that suggests age, sex, ethnicity, type of MS, duration of MS, and disability level are potentially non-modifiable predictors of PA (12). Similarly, in agreement with previous research, self-efficacy, fatigue, quality of life, employment status, participation, and autonomy may be important and potentially modifiable factors for modulating PA.

Although these findings suggest specific subgroups of people and potential modifiable factors to target to increase PA in this population, the majority of these factors were not associated with PA, when other non-modifiable and modifiable factors were controlled for. Therefore, although they are important to consider when developing and implementing PA interventions, they should not be considered in isolation. Walking capacity and the type of MS were the only independent correlates of PA. Exploring the barriers and facilitators to PA according to type of MS may inform development of PA interventions. Further, identifying strategies to improve walking capacity and supporting people with MS to engage in a variety of types of PA should be considered in future interventions to increase PA.

## Data Availability Statement

The raw data supporting the conclusions of this article will be made available by the authors, without undue reservation.

## Ethics Statement

The studies involving human participants were reviewed and approved by the College of Health and Life Sciences Research Ethics Committee in Brunel University London. The patients/participants provided their written informed consent to participate in this study.

## Author Contributions

JF and JR conceived the idea and analyzed the data. MN, AS, CK, GL, WH, CV, and LD contributed to the writing and assisted with the interpretation. JF completed this work while working at Brunel University London. All authors have read and approved the final manuscript.

## Funding

This work was supported by the MS Society UK (grant number 53).

## Conflict of Interest

The authors declare that the research was conducted in the absence of any commercial or financial relationships that could be construed as a potential conflict of interest.

## Publisher's Note

All claims expressed in this article are solely those of the authors and do not necessarily represent those of their affiliated organizations, or those of the publisher, the editors and the reviewers. Any product that may be evaluated in this article, or claim that may be made by its manufacturer, is not guaranteed or endorsed by the publisher.
