# Supplementary material for: Correlates of Objectively Measured Physical Activity Among People With Multiple Sclerosis: A Cross-Sectional Study
Source: Front Rehabil Sci. 2021 Dec 10;2:726436. doi: 10.3389/fresc.2021.726436 (PMC9397718; doi:10.3389/fresc.2021.726436)
Supplement: Supplementary file 1 [file Table_1.DOCX]

Table 1 Supplementary table 1

| Name | Purpose | Structure | Min-max |
| --- | --- | --- | --- |
| MFIS total score | Impact of fatigue on a person’s activities | 21 items (0-4) | 0-84 (higher =more fatigue impact) |
| MMSE control subscale | Confidence with managing symptoms and coping with demands of illness | 9 items (10-100) | 90-900 (higher=more confidence) |
| MMSE function subscale | Confidence with regard to functional abilities | 9 items (10-100) | 90-900 (higher=more confidence) |
|  |  |  |  |
| MSWS-12 total score | Impact of MS on a person’s walking capability over the past 2week | 12 items (1-5) | 12-60, converted to percentage (higher % = poorer walking capability) |
| MSIS-29 physical | Physical impact of MS | 20 items (1-5) | (0-100) higher = indicates greater impact of disease on daily function |
| MSIS-29 psychological | Psychological impact of MS | 9 items (1-5) | (0-100) higher = indicates greater impact of disease on daily function |
| EuroQol-5D-5L | Health-related Quality of life | 5 items (1-5) | Higher = better health state |
| IPA: autonomy indoors | Participant and autonomy indoors | 7 items (0-4) | 0-28 (higher=worse autonomy and participation) |
| IPA: family role | Family participation and autonomy | 7 items (0-4) | 0-28 (higher=worse autonomy and participation) |
| IPA: autonomy outdoors | Participation and autonomy outdoors | 5 items (0-4) | 0-20 (higher=worse autonomy and participation) |
| IPA: social life and relationships | social participation and autonomy | 7 items (0-4) | 0-28 (higher=worse autonomy and participation) |
